# Investigating the Significant Individual Historical Factors of Driving Risk Using Hierarchical Clustering Analysis and Quasi-Poisson Regression Model

**DOI:** 10.3390/s20082331

**Published:** 2020-04-19

**Authors:** Hasan A.H. Naji, Qingji Xue, Ke Zheng, Nengchao Lyu

**Affiliations:** 1 School of Computer and Information Engineering, Nanyang Institute of Technology, Chang Jiang Road No 80, Nanyang 473004, China; hasanye1985@gmail.com (H.A.H.N.); zhengke@nyist.edu.cn (K.Z.); 2 Intelligent Transport Systems Research Center, Wuhan University of Technology, Wuhan 430063, China; lnc@whut.edu.cn

**Keywords:** near-crash frequency, historical driver risk, hierarchical clustering analysis, quasi-Poisson regression model

## Abstract

Driving risk varies substantially according to many factors related to the driven vehicle, environmental conditions, and drivers. This study explores the contributing historical factors of driving risk with hierarchical clustering analysis and the quasi-Poisson regression model. The dataset of the study was collected from two sources: naturalistic driving experiments and self-reports. The drivers who participated in the naturalistic driving experiment were categorized into four risk groups according to their near-crash frequency with the hierarchical clustering method. Moreover, a quasi-Poisson model was used to identify the essential factors of individual driving risk. The findings of this study indicated that historical driving factors have substantial impacts on individual risk of drivers. These factors include the total number of miles driven, the driver’s age, the number of illegal parking (past three years), the number of over-speeding (past three years) and passing red lights (past three years). The outcome of the study can help transportation officials, educators, and researchers to consider the influencing factors on individual driving risk and can give insights and provide suggestions to improve driving safety.

## 1. Introduction

Motor vehicles have become the most popular transportation means in China. Every year over 20 million motor vehicle crashes happen, and billions of yuan are lost due to damage. Motor vehicle crashes are one of the leading causes of death, with over ten thousand fatalities annually in China [1]. To understand the reasons behind crashes, traffic safety research has become a widely recognized, highly invested in, and flourishing research area. Although vehicle crashes generally have various causes, it is commonly confirmed that human factors are one of the essential elements in vehicle crash causation [2]. Drivers may particularly be at high risk of being involved in a crash if they frequently adopt ill-suited behaviors when they are driving. In addition, risky driving behaviors have been considered as part of a method for characterizing driving style [3,4,5]. Much research and various methods have been proposed for investigating risky driving behaviors to prevent and reduce road crashes and accidents.

However, the main question as to how to effectively evaluate performance and driving risk at an individual level is still an active and challenging research topic for the following reasons.

Firstly, there is a lack of a comprehensive studies considering driving behavior and individual historical factors of drivers. This problem is addressed in large-scale naturalistic driving studies [4,5] where instrument vehicles are used to continuous collecting data to implement risk-assessment methods. In contrast, no information can be found considering the impact of historical driving factors, such as the number of incidents of speeding, which may provide insight into the risky driving style of drivers. Secondly, how to effectively and efficiently classify drivers and identify high-risk drivers before crashes happen is needed to be addressed. For instance, reference [6] used the K-means clustering method to group drivers according to their risky driving behavior. On the other hand, K-means requires the number of groups to be determined before performing classification, which may provide inaccurate results based on various selections of K. Identifying drivers’ classifications is a requirement in traffic safety-related applications. Thirdly, the lack of investigation of contributing individual historical factors correlated to driving risk. This is a challenging task as there is a demand for selecting and implementing the suitable regression model for the collected driving data to determine the essential factors which have significant impacts on individual driving risk.

In this publication, we investigate the contributing factors associated with individual driving risk considering historical driving factors using a dataset gathered using naturalistic driving experiments and a self-report method. Our focus is on characterizing driver behavior actions such as time headway, braking pressure, and acceleration in order to generate near-crash events. Then, near-crashes are used as safety-related events for driver classification. Furthermore, the hierarchical clustering method and the quasi-Poisson model are applied in driver classification to explore the contributing factors related to driver risk.

Specifically, the main contributions of the paper are summarized as follows: (1) providing a comprehensive study considering driving behavior factors and individual historical factors of drivers to evaluate driving risk. (2) The hierarchical clustering method is adopted to classify the drivers and to determine the high-risk drivers. (3) A quasi-Poisson model is performed to explore the contributing factors related to individual driver risk. (4) The proposed study has been implemented on a dataset collected from a naturalistic driving experiment and a self-reported method.

The rest of this paper is structured as follows. In Section 2, we introduce related work. Section 3 describes the naturalistic driving experiment and data collection methods. The proposed method is introduced in Section 4. In Section 5 we present the corresponding results in detail, followed by evaluation and discussion in Section 6. Finally, the conclusions and recommendations for future work are presented in Section 7.

## 2. Related Work

Extensive effort has been made in investigating parameters with a high impact on driving risk. Here is a summary of related important research aspects.

As drivers may particularly be at high risk of being involved in a crash if they frequently adopt ill-suited behaviors when they are driving, studying and analyzing driving behavior has become a hot topic. Wu et al [7] proposed a clustering-based method for classifying drivers by considering several typical driving behavioral characteristics based on GPS data mining.

Using this method, the drivers can be grouped according to their driving inclinations within the larger context of increasing traffic safety. Lyu et al [8] evaluated traffic safety by studying drivers’ behavior and performance under cognitive workload in complex environment areas. The study inferred that speed maintenance and lane deviations along with driving experience and gender groups may play an important role in analyzing and improving traffic safety in highway environments. Some research has analyzed the driver’s workload of physiological signal [9,10]. In [9], the authors investigated the psychological effects of stress and workload on drivers’ behavior during automated driving. The study [10] proposed a method for assessing the driver’s workload in real context and for obtaining the objective index of the drivers’ experienced workload to provide a potential reliable online trigger. Driver behavior plays a vital role in driver risk evaluation; however, it may be challenging to directly apply this to real-world driving.

A considerable number of studies have been conducted in evaluating individual driving risk [11,12,13]. Identifying factors related to individual driving risk can reduce the crash likelihood of the high-risk factors and can enhance overall driving safety. The individual driver’s risk can be influenced by many factors in many aspects. Moreover, demographic factors such as age, driver personality, and gender also play essential roles in individual driving risk [14]. Studies have shown that there is a connection between risky driving behavior and personality characteristics [15,16]. However, no study has considered the association between historical driving factors and risky driving behavior. Factors such as the number of times someone has passed through a red light and the number of incidents of speeding and illegal parking can highlight the driving style of drivers and help in analyzing drivers’ risk when they are behind the wheel.

Many studies have given valuable insights into the factors that influence the likelihood of a driver being in a vehicle crash. These studies generally used official traffic accident data, which in turn have two main limitations: (1) shortage of detailed driving data, and (2) challenge to collect and acquire (usually gathered by official traffic agencies) [17]. Thus, naturalistic driving studies (NDSs) provide a better choice to collect data for analyzing the risk of driving behavior [18,19,20]. The collected data present an alternative tool to link driver behavior with the individual driver risk. Naturalistic driving studies can gather rich kinematic, radar, global positioning system (GPS), and video related data at a high level of frequency, which allows abnormal driving circumstances to be exposed. For instance, the research project named “100-Car Naturalistic Driving Study” utilized a large-scale and well-prepared instrumented vehicle to gather naturalistic driving data in the United States [21]. However, the data collected by NDSs may not be enough to comprehensively analyze a driver’s risk; there is a need to collect and use variables for describing the historical risk factors of drivers.

Statistical models have been extensively adopted to understand and evaluate driving behavior risk. Wang et al [22] adopted the classification and regression tree model (CART) to study the association among driving risk, driver/vehicle characteristics, and road environment. Naji et al. [23] used the mixed-ordered logit model for evaluating driving risk based on near-crashes. The model was used to examine the contributing factors associated with the driving risk of near-crash events. The findings indicate that various variables influence driving risk including vehicle kinetic energy, deceleration average, near-crash causes, time of day, Total of Driven Mileage, road congestion, type of road, driver age, and driving experience. Guo et al. [24] used a negative binomial regression model to investigate the significant factors influencing the individual risk of drivers. The model investigated whether critical-incident events (CIEs) marked by high deceleration/acceleration or kinematic variables can be utilized to identify high-risk drivers. Moreover, the K-means clustering method is used to group the drivers into three groups. Factors such as age, CIE rate, and personality had considerable influence on individual driver risk. Although the statistical models above play a vital role in traffic safety assessment, some statistical models may not be perfectly used in driving risk evaluation. For instance, Poisson-based models such as a quasi-Poisson regression model can perform better than a negative binomial regression model and provide better findings on crash frequency analysis, which indicates that it is necessary to select the proper statistical model in such types of research.

Our method adopted a hierarchical clustering analysis and quasi-Poisson regression model for assessing significant individual historical factors of driving risk. Compared to previous approaches [22,24], our method used a quasi-Poisson regression model to explore the contributing factors related to individual driver risk. Like [19] and [22], a naturalist study was conducted to collect data; however, self-reported data of historical factors was gathered using a questionnaire. Furthermore, unlike [23,24], hierarchical clustering was used to classify drivers according to their driving risk level. Although the near-crash concept is used as a surrogate measure to evaluate the safety impact of driving behavior in this study, as in [22,24], the parameters used for near-crashes are different, namely braking pressure, time headway, and deceleration. These parameters can better describe driver risk when drivers are behind the wheel.

More specifically, the goal of this study was to make use of the collected naturalistic driving data along with self-reported data to investigate the association of historical individual driving factors and their impacts on driving risk. A quasi-Poisson regression model was conducted on the collected data to explore the contributing factors related to individual driver risk. Moreover, the hierarchical clustering method was adopted to classify the participant drivers efficiently and to determine the high-risk drivers.

## 3. Experiment Design and Data Collection

To provide a robust base for evaluating individual drivers’ risk, two components are vital for this study, namely experiment design and data collection.

### 3.1. Experiment Design

#### 3.1.1. Participants

In the study, a total of 41 drivers were recruited for the driving experiments, including 30 male drivers (73.17%) and 11 female drivers (26.82%). Participants’ age ranged between 18 and 56 years old, and the average was 31.85 (SD = 8.23). On the education level variable, 13.4% of the participants were in the “high school or below” group, 44.5% of them had an undergraduate degree, and 42.1% of them obtained a postgraduate degree or above. Regarding driving experience, 39.2%, 42.4%, and 18.4% had driving experience of “less than three years” “between three and ten years” and “more than ten years,” respectively. Regarding driving distance, 38.2% of the recruited drivers drove less than 20,000 km, 56.3% reported a number between 20,000 and 200,000 km, and 6.5% of the participants reported they drove more than 200,000 km.

To distinguish the driving features of the participants, they are numbered from 1 to 41. Table 1 provides more details about the drivers [23].

#### 3.1.2. Experiment‘s Vehicle

To collect driving data, naturalistic driving experiments were conducted using a well-prepared instrumented vehicle that was operated on varied types of roads of the city of Wuhan. The vehicle has been instrumented with various equipment. Table 2 summarizes the types of equipment and the collected data.

#### 3.1.3. Experiments Routes

**Figure 1:**
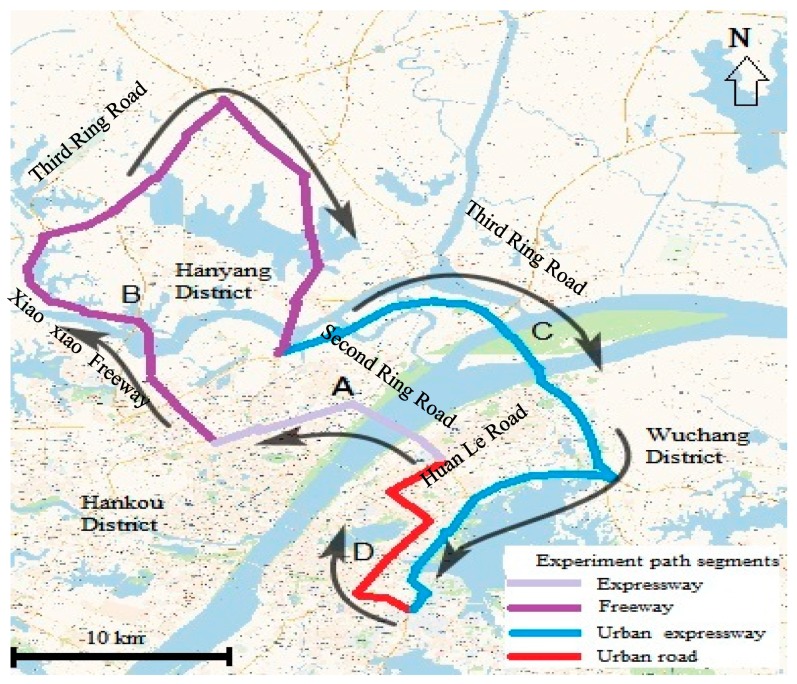
Whole experiment route on various road types of the city of Wuhan.

To consider the influence of different road types on driving behavior, a driving experiment path was designed to contain all different road types, and the total length of the planned route was 90 km, which took 90 min as the average driving duration. Figure 1 shows the whole experiment route on various road types of the city of Wuhan.

As can be seen in Figure 1, the whole experiment path consisted of four segments, namely A, B, C, and D. Segment A presents a 10 km length expressway on which the speed limit was 80 km/h. Segment B depicts a freeway that has 3–4 lanes in two directions, on which the speed limit ranged from 100 to 120 km/h, and the total freeway length was 38 km. The speed limit in Segment C, which represents an urban expressway, was 80 km/h, and the total distance was 31 km. Segment D depicts an urban road, which consists of two or three lanes in two directions, on which the speed limit was between 40 and 60 km/h and with a distance of around 12 km. The total length of the designed route was 90 km, which took 90 min as the average driving duration. The main features of the road segments driven during the driving experiments are listed in Table 3.

In order to guarantee the accuracy and quality of the obtained data, the participants performed an experiment test for the necessary process of the experiments to be ready for the experiments. During the experiments, each participant drove the experiment vehicle on different types of roads on the determined route. Each experiment was conducted at different times of day ranging from 8:00 to 20:00. Moreover, route guidance was given to the participants using a navigation map in the vehicle during the driving experiments.

### 3.2. Near-Crash Events

Recently, many researchers began paying attention to the possibility of using near-crashes to study the contributing factors related to risky driving. For instance, Guo and Fang [24] introduced a method for evaluating an individual driver’s driving risk using naturalistic driving data.

A near-crash indicates that a driver performs a sudden evasive action (i.e., emergency braking and steering operation), without such an action, a real crash may occur [24]; thus, previous studies provide various definitions of a near-crash event. Wu et al. [25] focused on braking events and considered the occurrence of a near-crash event where braking is the primary evasive maneuver. Wang et al. [22] detected a near-crash event when the vehicle’s acceleration reached a predetermined threshold (longitudinal: −1.5 m/s^2^, lateral: −1 m/s^2^).

To better understand the driving risk of naturalistic driving, this study adopted a definition of near-crash by considering significant variables of driving behavior. Three essential variables, acceleration, braking pressure, and time headways, are considered, as in reference [23]. During a naturalistic driving experiment, a near-crash event is identified by detecting one (or more than one) of the three thresholds of driving signals as follows: (1) acceleration (ACC) is less than −0.4 m/s^2^, (2) time headway (THW) is less than 0.6 s, or (3) braking pressure is more than 10 mpa.

### 3.3. Data Analysis

To provide a build affirm foundation for exploring the individual risk of drivers, this study used the obtained data from the naturalistic driving experiments was used along with the self-report data collected from participants.

Traffic surveys showed that the bigger the increase of some variables, such as speed average and acceleration average, the higher the risky the accident would be, which may increase the probability of an accident occurring [7,25]. Therefore, for the aim of initially comparing the differences of driving risk among the participants, box-line figures were plotted to depict the dispersion of driving performance of each driver according to the collected data from naturalistic driving experiments.

**Figure 2:**
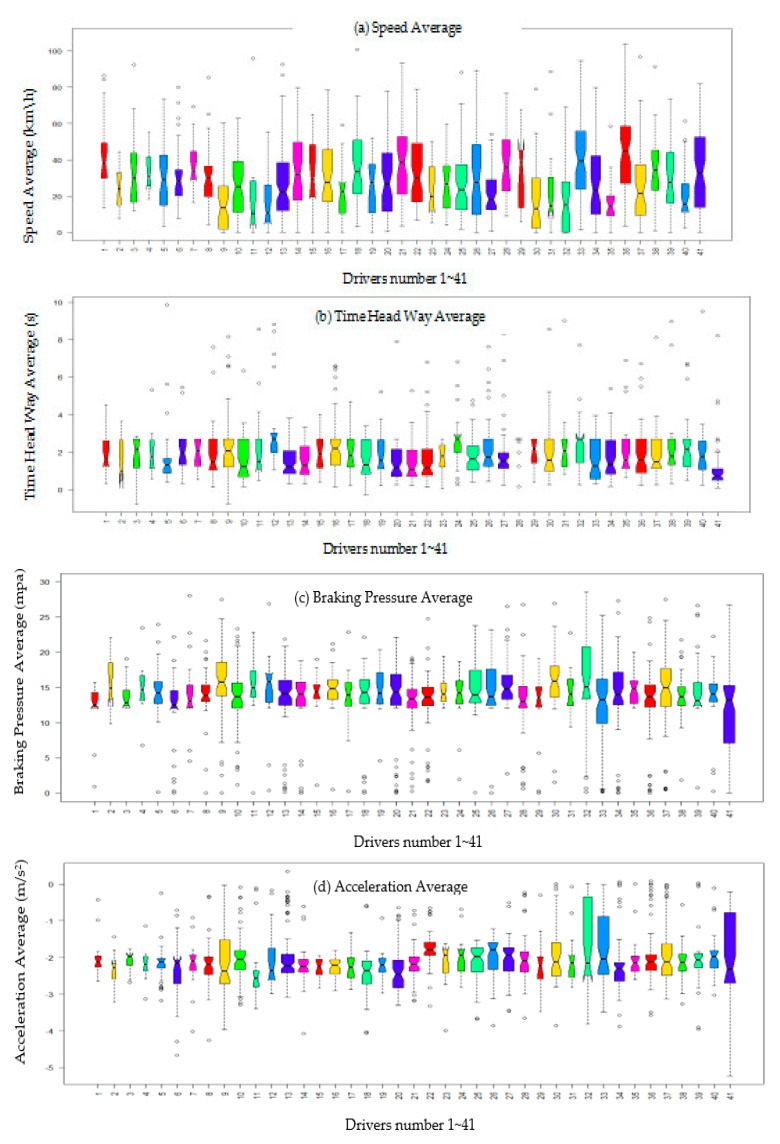
Box plot analysis of driving behavior of experiment participants. (**a**) Speed Average, (**b**) Time Head Way Average, (**c**) Braking Pressure Average, (**d**) Acceleration Average.

Figure 2 shows the distribution of speed average, a time headway, acceleration average, and braking pressure of each driver.

As shown in Figure 2a, the speed average distribution of participants with driving behavior is different. The median line and the upper quartile line of drivers 41, 36, 26, 21, and 33 are located in the range [25, 80], which is higher than that of drivers 40, 32, 12, 11, and 9, who were in the area [0, 25]. Regarding time headway average, the lower quartile and median of drivers 41, 33, 13, 9, 19, 20, and 21 are in the lower area of the scale [−0.5, 2], which is lower than that of drivers 12, 24, 40, 5, 11, 38, and 27, who are distributed in the upper interval [2, 10]. Figure 2c shows that the median and upper quartile line of drivers 41, 22, 37, 32, and 33 lie at the upper level [15, 25], which is higher than that of drivers 28, 17, 12, 32, and 33, located at the lower level [0, 15]. Finally, the acceleration average distribution of the box plot of drivers 41, 32, 33, and 22 is much higher than that of other drivers, such as drivers 6, 9, 12, and 17, located in the lower area [−5,−2.5].

To sum up, from the details obtained from Figure 2, we can initially infer that drivers 41, 33, 21, and 32 are considered as high risk drivers, whereas 9, 11, 12, and 17 are considered as conservative drivers.

From the explanations above, we can understand the vital role that these variables can play in assessing driving risk by using the generation of near-crash events. Therfore, as in Section 3.2, near-crash events were used as a surrogate measure of driving risk.

The frequency of near-crash events of each driver who participated in the experiments are considered. Moreover, through the self-report method, more variables could be obtained. Finally, nine variables of individual driving factors were considered as well. Table 4 provides a summary description of the variables of individual driver risk.

It is necessary to mention that due to the extreme value variation of some variables such as total driving mileage, a normalization process is required. The normalization equation is conducted using Equation (1):

(1) $$x^{*}=\frac{x-x_{avr}}{x_{max}-x_{min}}$$

where *x_avr_*, *x_max_*, *x_min_* are the average, maximum, and minimum of a variable. The driving experiments resulted in near-crash events of forty-one participants. The rate of near-crash events of each driver *i* was calculated as in Equation (2):

(2) $$\mathrm{Near}\;\mathrm{crash}\;\mathrm{rate}\left(i\right)=\frac{\mathrm{Frequency}\;\mathrm{of}\;\mathrm{near}\;\mathrm{crashes}\;\left(i\right)}{\mathrm{Driven}\;\mathrm{Miles}\left(\mathrm{per}\;1000\;\mathrm{miles}\right)\left(i\right)}$$

## 4. Methodology

In our study, near-crash frequency is considered as the main criterion for examining an individual driver’s risk. Thus, two main aims are considered: classifying drivers based on their driving risk and investigating risk factors related to individual driving. Therefore, firstly, hierarchical clustering analysis was adopted for grouping and classifying high-risk drivers. Secondly, a quasi-Poisson regression model was used to determine the significant factors which have a substantial impact on individual driving risk. The details of the clustering analysis technique and regression models are discussed in this section.

### 4.1. Hierarchical Clustering Analysis

Recently, many studies in traffic safety have adopted cluster analysis as the proper approach to group and identify drivers into different groups [7,26]. K-means, Density-Based Spatial Clustering of Applications with Noise (DBSCAN) and hierarchical clustering are popular clustering techniques used in traffic safety research. 

The hierarchical clustering method has become commonly used in many studies for categorizing objects in a multiple-level hierarchy. Unlike the K-means method, the number of clusters (K) is not required. Hierarchical clustering has two main methods, namely agglomerative and divisive. In both methods, there are N-1 levels in the hierarchy model [27]. In the agglomerative method, smaller clusters are formed first, and a combination of the clusters is performed to make larger clusters. On the other hand, in the divisive method, a bigger cluster is formed initially, followed by dividing it into sub-clusters.

In this study, hierarchical clustering was used to group drivers into clusters by taking their near-crash rate into consideration, which in turn resulted in a tree-like structure called a dendrogram. Firstly, a linkage criterion and a distance measure (e.g., Euclidean distance) were utilized to implement the algorithm. In the agglomerative method, the algorithm starts with no clusters, and then each driver is considered as a separate cluster. During the algorithm implementation process, each of the two most similar clusters are combined, and the algorithm is terminated in the case that all drivers formed a single cluster. The distance measure in the algorithm can identify the association between the drivers of the study by determining the calculating method of the similarity among the drivers’ near-crash rates, which are represented visually by points. Euclidean distance [27], the square root of the sum of the squared differences, is the most popular measurement used in the hierarchical clustering method. Equation (3) calculates the distance between two near-crash rates (*x* and *y*) using an Euclidean distance measure, where the variable *p* is the total number of near-crash events.

(3) $${\mathrm{dist}}_{\mathrm{Euclidean}}\left(x,y\right)=\sqrt{\sum_{i=1}^{p}{\left(x_{i}-y_{i}\right)}^{2}}$$

**Figure 3:**
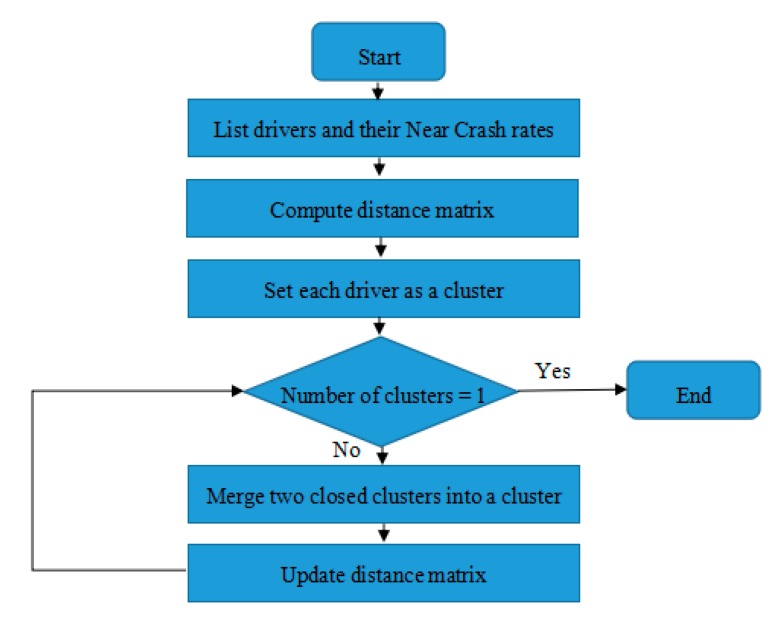
The steps of clustering algorithm for grouping drivers.

Figure 3 illustrates the steps of the clustering algorithm for grouping drivers.

### 4.2. Regression Model

As mentioned above, the goal of the study was to evaluate the impact of individual historical risk factors on driving risk by examining the significant factors of near-crashes by taking into consideration the effect of the outcome variable value (near-crash frequency of drivers).

In order to avoid misleading or skewed results, a test for the existence of the collinearity among the variables was conducted using a car package in R software. The variance inflation factor (in short VIF) was calculated by function VIF. The interpretation of VIF is as in Equation (4):

0 < VIF < 10      No Collinearity 10 ≤ VIF < 100      Strong Collinearity VIF ≥ 100          Serious Collinearity(4)

For the sake of understanding the nature and distribution of the dependent variable (near-crash frequency), the relation between the variance and the mean is examined. We found that the dependent variable’s variance is larger than the mean, which is defined as over-dispersion [28].

A common way to deal with over-dispersion counts is to use generalized linear models, including the quasi-Poisson model, the negative binomial model, or the zero-inflated Poisson model [29]. The following is an overview of those statistical models, along with related formulas.

#### 4.2.1. Quasi-Poisson Model

In the quasi-Poisson regression model [30], the variance is calculated by multiplying the mean with a specific dispersion parameter. Thus, the quasi-Poisson model considers over-dispersed data, which is a general characteristic of traffic-related data, such as numbers of crashes. 

Assume *NCF*_1_, *NCF*_2_, …, *NCF_n_* represent the near-crash frequency of a driver, *i*, and *μ_i_* is the mean such that

*μ*_i_ = E(*NCF_i_*) (5)

In the following analysis, a function of log link connects *μ_i_* with a set of covariates *x_i1_*, *x_i2_*, …, *x_ip_*:

(6) $$\left(\mu_{i})=\log(\mu_{i})=\sum_{j=1}^{p}x_{ij}\times \beta_{j}+\log(d_{i}\right)$$

where *d*_i_ is the near-crash exposure measure (distance traveled per 1000 miles) for a driver *i*. The logarithm of *d*_i_ above will be an offset term with a fixed coefficient of one under the log link function.

Instead of considering assumptions on the distributions on near-crash data, quasi-likelihood models only need a specification of the relationship between the data mean and the variance [27]. In addition, the quasi-Poisson model uses the association from the Poisson distribution, thus the variance, Var, is related to the mean, *μ_i_*, only using the multiplication of the dispersion, *ϕ*, as follows:

(7) $$\mathrm{Var}(NCF_{i})=\phi V(\mu_{i})=\phi \mu_{i}$$

where Var is the variance function which describes how the variance of *NCF_i_* relates to its mean. The quasi-score function q(*μ*_i_,*η_i_*,*ϕ*) is obtained by the first-order derivative of the log likelihood function, which adopts the same calculation method as the traditional score function of the generalized linear model. In the quasi-poison model, calculating the score function for a single observation, *i*, is as follows:

(8) $$q(\mu_{i},\;\eta_{i},\phi )=\frac{\eta_{i}-\mu_{i}}{\phi V(\mu_{i})}=\frac{\eta_{i}-\mu_{i}}{\phi \mu_{i}}$$

where $n_{i}$ is a sample value for the number of near-crash events of a driver, *i*. Thus, the quasi-likelihood function for a simple *i* can be written as follows:

(9) $$Q(\mu_{i},\eta_{i},\phi )=\int_{\eta_{i}}^{\mu_{i}}q(\mu_{i},\eta_{i},\phi )ds=\int_{\eta_{i}}^{\mu_{i}}\frac{\eta_{i}-s}{\phi s}ds$$

Finally, the quasi-likelihood’s function for all samples is the summation of the quasi-likelihood’s function for every observation, as Equation (10):

(10) $$Q(\mu ,\eta ,\phi )=\sum_{i=1}^{m}Q(\mu_{i},\eta_{i},\phi )ds=\sum_{i=1}^{m}\int_{\eta_{i}}^{\mu_{i}}\frac{\eta_{i}-s}{\phi s}ds$$

The estimated parameters will maximize the value of Q(*μ*, *n*, *ϕ*), and the estimation’s equation is as the following equations:

(11) $$\frac{\partial Q}{\partial \beta }=\sum_{i=1}^{m}q(\mu_{i},\eta_{i},\Theta )\frac{\partial \mu_{i}}{\partial \beta }=\sum_{i=1}^{m}\left(\frac{\eta_{i}-\mu_{i}}{\Theta \mu_{i}}\right)\frac{\partial \mu_{i}}{\partial \beta }=0$$

which is equivalent to

(12) $$\sum_{i=1}^{m}(\eta_{i}-\mu_{i})\times \sum_{j=1}^{p}x_{ij}\times \beta_{j}$$

Furthermore, in terms of the regression’s parameters *β* and exposure variable *t*, the system of Equation (13) [31] is also as follows:

(13) $$\sum_{i=1}^{m}(\eta_{i}-t_{i}\exp(\sum_{j=1}^{p}x_{ij}\times \beta_{j}))=0$$

#### 4.2.2. Negative Binomial Model

The negative binomial regression model is popular for traffic safety modeling [32]. In this section, we only list the main related equations, and for model derivation see reference [33].

Negative binomial model assumes that the near-crashes frequency for a driver, *i*, NC_Fre_i_, follows a negative binomial distribution as

NC_Fre_i_=NB(NC_Rate_i_, *d*_i_, γ) (14)

where NC_Rate_i_ is the near crash rate of the driver, *i*, *d*_i_ represents the miles traveled by the driver *i* (during the experiment), and γ is the over-dispersion parameter of the negative binomial distribution. The log link function Log(*d*_i_) connects the traveled miles, *d*_i_, with a set of covariates, as in Equation (15):

Log(*d*_i_) = *X_i_* × *β* (15)

where *X_i_* is the vector of variables for driver *i* and *β* is the vector of the negative binomial regression parameters. In our study, age, gender, driving license period, total driving mileage, driving mileage (last year), number of accidents (past three years), number of illegal parking incidents (past three years), number of speeding incidents (past three years), and number of incidences of going through a red light (past three years), were used as the vector of variables X of the driver *i*.

#### 4.2.3. Zero-Inflated Poisson (ZIP) Model

For the aim of briefly introducing the ZIP model as defined in the [34], let Y*_i_* be the ZIP random variable, which in this study is the frequency of near-crash (NC_Fre) of the driver *i*. The probability mass function (pmf) of *Y* is:

(16) $$P\left(Y=y\right)=\{\begin{array}{c} \omega +\left(1-\omega \right)e^{-\eta },\;\;\;y=0,\\ \left(1-\omega \right)\frac{e^{-\eta }\eta^{y}}{y!},\;\;y>0 \end{array}$$

where *y* = 0, 1, 2, …, *η* is the Poisson mean, and *ω* ∈ [0, 1] is the mixing probability parameter to accommodate the extra zeros. Once *ω* = 0, Equation (16) will be reduced to a general Poisson distribution, which is:

(17) $$P(Y=y|\omega =0,\eta )=\frac{e^{-\eta }\eta^{y}}{y!},y=0,1,2,\ldots ,\eta$$

The mean μ(Y) and the variance Var(Y) of a ZIP random can be computed as follows:

μ(Y) = (1 − *ω*)*η* (18)

Var(Y) = (1 − *ω*)*η*(1 − *ωη*) (19)

As we can see, Poisson distribution is not useful as Var(Y) > μ(Y).

In practice, ω is linked to a set of dependent variables (X) via a logit linear predictor. Technically, *ω* = H(u) = H(*β^T^X*), where H(u) = [1 + exp(−u)] − 1 [34].

Similarly, *η* is linked to set of dependent variables (X) via a log-linear predictor *η* = exp(*γ^T^X*) for unbounded count data.

Following [35], the likelihood function of the Zero-Inflated Poisson model is:

(20) $$L(\theta )=\left.\begin{array}{l} ({\prod_{i=1}^{n}\left[\frac{H(\gamma^{T}X_{i})}{H(\gamma^{T}X_{i}+\exp(\beta^{T}X_{i}))}\right]}^{I(Y_{i}=0)})\;\times \\ \left({\prod_{i=1}^{n}\left\{\left[1-H(\gamma^{T}X_{i})\right]\left[\frac{\exp\left[-\exp(\beta^{T}X_{i})\right]{\left[\exp(\beta^{T}X_{i})\right]}^{Y_{i}}}{Y_{i}!}\right]\right\}}^{I(Y_{i}>0)}\right) \end{array}\right.$$

The log-likelihood function of a ZIP model is

(21) $$\zeta (\theta )=\log\;\Theta (\theta )=\sum_{i=1}^{n}\zeta_{i}(\theta )$$

By substantiating in Equation (20), *ω_i_* = *H* (*β^T^X_i_*) and *η_i_* = exp(*γ^T^X_i_*) since *i* = 1,2, …, n and θ = *ω* = (*β^T^_,_γ^T^*)*^T^* is a vector of the unknown parameters of the regression of the interest that needs to be estimated [35].

### 4.3. Model Evaluation

To specify the most suitable regression model among the three generalized linear models above (quasi-Poisson, negative binomial, and zero-inflated Poisson model), there is a need to evaluate the models according to the Akaike information criterion (AIC) [36] and Bayesian information criterion (BIC) [37].

By using the maximum-likelihood method, the AIC and BIC are utilized for model selection. AIC and BIC are formally defined as Equations (22) and (23) as follows:

AIC = −2log p(y|β_mle_) +2k (22)

BIC = −2log p(y|β_mle_) +k log(2) (23)

where p(y|β_mle_) is the likelihood function and k is the number of parameters [38].

## 5. Results

### 5.1. Statistical Summary of Data

After collecting the dataset and variables using naturalistic driving experiments and the self-report method, there is a need to understand the overall view of the collected data before conducting the clustering analysis and regression process. Table 5 lists the basic descriptive statistics for explanatory variables along with the exposure variable.

### 5.2. Hierarchical Clustering Analysis

**Figure 4:**
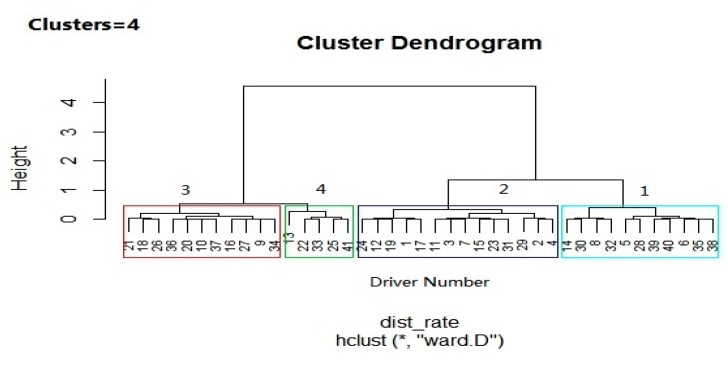
A cluster dendrogram classifying drivers based on driving risk (near-crash frequency). (1) “Conservative”, (2) “Normal”, (3) “Serious”, and (4) “Severe”.

By using Ward’s linkage method (and z-score standardization to eliminate bias in values), hierarchical dendrogram clustering was produced based on the results of near-crash rates of the participating drivers, as shown in Figure 4.

In Figure 4, the drivers (participants) are grouped according to their driving risk level into four clusters, namely “Conservative”, “Normal”, “Serious”, and “Severe” represented by numbers 1, 2, 3, 4, respectively.The conservative and normal clusters showed a low driving risk with 61% of the participants. The serious cluster was considered to be at a high risk driving with 24% of the participants, and the severe cluster was at very high risk, but only included 12% of the participants. It is evident that a very small number of drivers were found in the severe risk group (5 drivers), and this is reasonable and fits the context well.

In order to explore the variation of the individual driving risk of the four risk groups, Table 6 provides a statistical summary of characteristics of the four risk groups.

**Figure 5:**
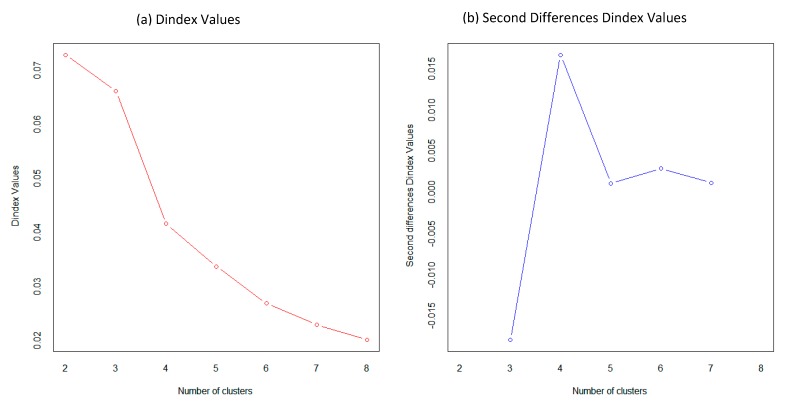
(**a**) Dindex Values (**b**) Second Differences Dindex Values for Selecting the optimal clustering number of driver clusters.

To obtain the optimal clusters’ number, we used the NbClust package in the R language. NbClust provides 30 indices for determining the number of clusters and proposes the best clustering scheme from the different results obtained by varying all combinations of the number of clusters, distance measures, and clustering methods. The Dindex is a graphical method of determining the optimal number of clusters. We generating the clustering process by changing the clustering number from 2 to 8 to determine the optimal clustering number, as shown in Figure 5.

In the plot of Dindex, we explored the significant knee (a considerable peak in Dindex that corresponds to a substantial increase of the value of the measure) and finally according to the majority rule, 4 was selected as the best number of clusters.

### 5.3. Model Estimation Results

The variables which had collinearity have been removed, such as near-crash rates. Then, the final results reported that the acquired maximum value of the VIF reached 7.1, and this value emphasizes that there is no collinearity found among the variables. Moreover, the likelihood ratio test (in short, LR test) was adopted to guarantee that all variables significantly improve the model’s overall performance.

For the goal of performing a comparison of model performance, besides the quasi-Poisson model, the negative binomial model and zero-inflated Poisson model (ZIP) were utilized as well. A comparison of the performance of the three models is introduced first, followed by providing interpretation for the estimates of the obtained parameters.

#### 5.3.1. Model Performance

Table 7 lists the results of the goodness-of-fit (GoF) measures of the quasi-Poisson model, the negative binomial model, and the zero-inflated Poisson model (ZIP). Incorporating the random parameters increased the model’s complexity but led to a significant enhancement in the overall fit as denoted by LL(β). In addition, the adjusted ratio index of log-likelihood, in which the higher values of a quasi-Poisson model denote that this model can explain the data better than the other models, is 0.315, which is higher than that of ZIP and negative binomial (0.233 and 0.315).

The results in Table 7 and Table 8 show that in this study, the quasi-Poisson model is more appropriate and outperformed the ZIP and negative binomial models. Therefore, in the following section, we list the estimation results of the quasi-Poisson model.

#### 5.3.2. Estimation Results of the Quasi-Poison Model

Table 8 lists the estimates of the factors from the quasi-Poisson model of driving risk using the frequency of near-crash events. Through the estimation, the variables with a value above 0.05 or above 0.1 were highlighted and considered significant, and other variables were considered to be non-significant and eliminated from the estimated results.

As shown in Table 9, we highlighted the values and signs of the estimated coefficient *β*. It is important to remember that the negative (or positive) value of coefficient *β_i_*, is related to an increase of the corresponding variable x*_i_*. This may decrease (or increase) the likelihood of high driving risk of an individual driver and may increase (or decrease) the likelihood of low driving risk as well.

In our study, the explanatory variables X which resulted in coefficients with positive signs were the number of incidents of illegal parking (past three years), speeding (past three years), and going through a red light (past three years). In other words, the drivers with a high number of illegal parking (past three years), speeding (past three years), and going through a red light events may increase the probability of them taking part in high-risk driving and being considered as serious and severe risk drivers. In addition, the drivers with a high total of miles driven (km) and age larger than 45 are likely to be a low driving risk and considered as conservative and normal risk drivers.

Moreover, Table 9 shows that the two most significant positive variables were associated with the number of illegal parking events (past three years) followed by the number of speeding events (past three years) (*β* = 0.4041166 and 0.2032453 respectively). It denotes that these variables are the most critical ones and may considerably increase the driving risk of individual drivers. By comparing the estimated coefficients of the variables in Table 9, we can rank the impact of all variables on driving risk. We found that number of occurrences of illegal parking variable has the greatest impact on driving risk (*β* = 0.4041166).

Furthermore, the variables with negative coefficients included the total number of miles driven (km) and age larger than 46. Moreover, the total number of miles driven was combined with the largest negative parameter listed in Table 9, which shows that high driving risk may decrease considerably once the total number of miles driven by the driver increases. It may be an asset that driving experience plays a vital role in enhancing traffic safety.

## 6. Discussion

Comparing our study to [22,24], we highlight the similarities and dissimilarities. Regarding the methodology and results, this study has the following differences and innovations.

Firstly, a comprehensive experiment was conducted on different types of roads in the city of Wuhan, China, to include the variables related to driving risk, which makes this study more comprehensive and reasonable. The collected variables include driving behavior, near-crash factors, demographic information of drivers, and historical individual driving factors. In contrast, significant variables were not included in the study [24], such as the number of incidents of going through a red light, speeding, and illegal parking, driving license period, and driven mileage. These variables indeed play a significant role in evaluating the driving risk of individual drivers, and some of these variables were considered significant according to the results of the study.

Secondly, this study adopted the definition of near-crashes proposed in [23], considering three variables: time headway, acceleration, and braking pressure. These variables provide powerful evidence of the considerable role that near-crashes can play on driving risk evaluation. The study in [17] only considered the braking variable for the definition of near-crash events.

Thirdly, [22] adopted the k-means method to group drivers based on driving risk into three levels (low, moderate, and high). In contrast, this study grouped the drivers using near-crash frequency by hierarchical clustering analysis. Hierarchical clustering analysis produced four clusters (conservative, normal, serious, and severe) that reasonably reflect the categories of drivers based on their driving risk.

Finally, Guo’s study [24] adopted a negative binomial model to explore the contributing factors to the driving risk of individual drivers. However, our study examined three models (negative binomial, zero-inflated Poisson, and quasi-Poisson) associated with over-dispersion data and finally adopted the quasi-Poisson model. In our study, the quasi-Poisson model achieves performance better than similar models. It resulted in more significant variables: the number of illegal parking (past three years), speeding (past three years), and going through a red light (past three years) events, the total of number of miles driven (km), and age over 45.

Individual driver risk was evaluated using the quasi-Poisson model to specify the individual historical factors (variables) associated with the frequency of near-crash events. The results showed that several significant factors such as age over 46, total number of miles driven, and number of illegal parking (past three years), speeding(past three years), and going through a red light (past three years) events, respectively, have significant impacts on the individual risk of drivers. The correlation between driver personality factors and driving risk were evaluated in previous studies [38,39]. A detailed description of the impact of the variables follows.

The driver demographic factor (age over 45) is considered a significant factor. It is interpreted that a driver who is over 45 years old shows a significantly lower driving risk due to driving experience than younger drivers who tend to drive fast and may commit driving errors, and this can lead to higher risk and causing crashes. This result conforms to the findings of the study in [38]. 

It is not surprising that the factors related to personal driving experience such as total driving mileage are considered as significant factors in driving risk. Unlike driving license period, total driving mileage represents the actual number of miles driven by drivers. As found in the quasi-Poisson model, the higher the value of driving mileage, the lower the near-crash rate will be. This is reasonable as the more miles that are driven, the more driving experience the drivers obtain, and this may lead to an increase in a driver’s safe driving [39].

The number of over-speeding events within the last three years is considered as a significant factor in this study. It is not surprising that the speeding variable is considered as one of the main causes of accidents and surely has a connection with increased driving risk [40].

An increased number of illegal parking (past three years) and passing through a red light (past three years) events can be considered as signs of an aggressive driving style, which can increase the ability and willingness to ignore regular traffic rules and may cause a driver to be involved crashes.

## 7. Limitations

Although the data used in this study includes various vital variables, there are three limitations. The first is that drivers might not be representative of all drivers since they were recruited for the experiments. The second limitation is the dataset used in this study does not include more information on the participants, such as education level, accommodation, sleeping condition, tiredness, job, etc. Such variables can potentially enhance the explanatory power of the results of the study. They can provide a more comprehensive understanding of different individual driver risks and help in presenting suggestions and recommendations to transportation officials. Moreover, the sample size was quite small (only forty-one drivers). A larger sample size could provide a more unobstructed view of the correlation between driving risk and individual driving factors.

## 8. Conclusions

This study investigated the contributing individual historical factors of driving risk using hierarchical clustering analysis and a quasi-Poisson regression model. Naturalistic driving experiments on various types of roads in the city of Wuhan, China, along with a self-report method, were conducted with forty-one participants to collect the required dataset for the study.

A near-crash event used as a surrogate measure of driving risk. Hierarchical clustering analysis was used to group drivers into clusters based on their near-crash frequency. Adopting hierarchical clustering analysis produced four clusters (conservative, normal, serious, and severe) that reasonably reflect the categories of drivers based on their driving risk.

A quasi-Poisson regression model was utilized to explore the significant individual historical factors of driving risk associated with near-crash frequency.

The results showed that several significant factors such as age larger than 45, total number of miles driven, the number of incidents of illegal parking (past three years), the number of over- speeding (past three years), and passing red lights (past three years) have significant impacts on individual driving risk.

The findings of the study can help transportation officials to determine the influencing factors on individual driving risk and can give insights and provide suggestions to improve driving safety and make roads safer.

For future work, there is a need to investigate the driving risk related to specific types of drivers, such as truck drivers and motorcyclists. Moreover, studying the impact of driving distractions, such as mobile phones and tiredness, should be considered as well.

## Figures and Tables

**Table 1:** Means and standard deviations of the background variables of recruited drivers.

	%	Total	Age (Years)		Driving Experience (Years)		Driving Distance (km)	
			Mean	SD *	Mean	SD	Mean	SD
All	100	41	31.85	8.23	6.7	4.49	266.44	113.4
Male	73.17	30	31.46	8.11	6.2	4.37	302.90	120.9
Female	26.82	11	33.00	8.74	8	4.83	158.90	137.2

SD* Standard Deviation.

**Table 2:** Equipment and collected data of naturalistic driving experiment.

Equipment	Collected Data
A GPS/INS	Vehicle’s Latitude and Longitude
CAN BUS	Speed, Brake Pedal, Accelerator Pedal, and Steering Wheel Angle
LiDAR	Velocity, Distance, Relative Velocity, Etc.
Mobileye	Lane Position, Time Headway, and Lane Departure
Video Camera	Environmental Information

**Table 3:** Main features of segments of experiment path.

	Segment A	Segment B	Segment C	Segment D
Road Type	Expressway	Freeway	Urban Expressway	Urban Road
Length (km)	10	38	31	12
Limited Speed (km/h)	80	100–120	80	40–60

**Table 4:** Summary description of variables of individual driver risk.

Variable	Symbol	Datatype	Description
Near-Crash Frequency	NC_Fre	Continues	Frequency of near-crash events
Near-Crash Rate	NC_Rate	Continues	Near crash frequency/1000 miles
Age	Age	Categorical	1. <23; 2. 23–45; 3. ≥45
Gender	Gender	Categorical	1. Male 2. Female
Driving License Period	Dri_lec	Continues	Driving license (years)
Total Driving Mileage	Dri_Mile	Continues	Total driving mileage (miles)
Driving Mileage (Last Year)	Dri_Mile2	Continues	Driving mileage last year (miles)
Number of Accidents (past three years)	Acc_no_3	Continues	Number of accidents in the past three years
Number of Illegal Parking (past three years)	Ill_Park	Continues	Number of illegal parking events in the past three years
Number of Over Speeding (past three years)	Over_Sp	Continues	Number of speeding events in the past three years
Number of Cross Red Light (past three years)	Cross_red	Continues	Number of passing through a red light events in the past three years

**Table 5:** Summary statistics of variables of the 41 participants.

Variable	Notation	Min	Max	Mean	SD	%Zero
Near-Crash Frequency	*y_i_*	10	87	40.73	19.62	4.87
Near-Crash Rates *	*x_i1_*	0.1	0.8447	0.399	0.19	2.43
Total Driven Mileage (km)	*x_i2_*	100	102.5	105	1.88	2.43
Age	*x_i3_* = 1: <23 = 2: 23 < *x_i3_* < 45 = 3: >45	-	-	-	-	-
Gender	*x_i4_* = 1: male = 2: female	-	-	-	-	-
Driving License Period (years)	*x_i55_*	2	18	6.92	4.66	4.87
Diving Mileage (10 km)	*x_i6_*	0.04	356	27.02	0.298	2.43
Diving Mileage Last Year	*x_i7_*	0	4	1.329	1.23	4.87
Number of Accidents (past three years)	*x_i8_*	0	4	1.112	1.21	7.31
Number of Illegal Parking (past three years)	*x_i9_*	0	1	0.371	0.25	2.43
Number of Over Speeding (past three years)	*x_i10_*	0	1	0.130	0.26	2.43
Number of Cross Red Light (past three years)	*x_i11_*	0	1	0.195	0.36	0

* See Equation (2).

**Table 6:** Comparison of hierarchical clustering results.

Symbol	Level	Number of Drivers	Percent	Drivers
1	Conservative	11	27%	14, 30, 8, 32, 5, 28, 39, 40, 6, 35, 38
2	Normal	14	34%	24, 12, 19, 1, 17, 11, 3, 7, 15, 23, 31, 29, 2, 4
3	Serious	11	25%	21, 18, 26, 36, 20, 10, 37, 16, 27, 9, 34
4	Severe	5	12%	13, 22, 33, 25, 41

**Table 7:** Goodness-of-fit measures for Zero-Inflated Poisson (ZIP), Negative Binomial, and Quasi-Poisson model.

Measure	ZIP	Negative Binomial	Quasi-Possion
Observations, n	41	41	41
Significant parameters, k	9	9	10
Log likelihood at zero, LL(0)	−306.064	−178.2979	−125.5942
Log likelihood at convergence, LL(β)	−229.229	−167.7601	−117.2174
Adjust likelihood ratio index	0.157	0.233	0.315
Degree of freedom	13	13	13

**Table 8:** **AIC and BIC** measures for ZIP, Negative Binomial and Quasi-Poisson models.

Model	−2loglik	AIC	BIC
ZIP	486.04	484.4594	506.7358
Negative Binomial	413.51	361.5202	383.7966
Quasi-Possion	410.42	260.4348	282.7112

**Table 9:** Estimate results of quasi-Poisson model.

Dependent Variable	Coefficient	Standard Error	Z-Statistic	p > |z|
Total of Driven Mileage (km)	**−0.0194187**	**0.0100464**	**−1.93**	**0.053** **
Age				
= 1 less than 23 ^a^	0	0	0	0
= 2 between 23and 45	0.0079925	0.0594635	0.13	0.893
= 3 larger than 45	**−0.1818544**	**0.1334087**	**−1.36**	**0.073** **
Gender				
= 1 Male^a^	0	0	0	0
= 2 Female	−0.0132278	0.0371807	−0.36	0.722
Driving License Period (years)	0.393108	0.0779334	0.50	0.614
Diving Mileage Last Year	−0.0003479	0.0198312	−0.02	0.986
Number of Accidents (past three years)	0.0123625	0.0145658	0.85	0.396
Number of Illegal Parking (past three years)	**0.4041166**	**0.1049027**	**1.95**	**0.052** **
Number of Over Speeding (past three years)	**0.2032453**	**0.1059235**	**1.92**	**0.055** **
Number of Cross Red Light (past three years)	**0.1627889**	**0.0560032**	**2.91**	**0.004** *
**Threshold**	**Coefficient**	**Standard Error**		
Cut-point	0.6181954	1.020766		

* Significant at 5% level. ** Significant at 10% level. ^a^ Base reference of an associated categorical variable. Bold numbers indicate significant variables.
